# Cytokine-Induced Modulation of SARS-CoV2 Receptor Expression in Primary Human Nasal Epithelial Cells

**DOI:** 10.3390/pathogens10070848

**Published:** 2021-07-05

**Authors:** Mahnaz Ramezanpour, Harrison Bolt, Karen Hon, George Spyro Bouras, Alkis James Psaltis, Peter-John Wormald, Sarah Vreugde

**Affiliations:** 1 Department of Surgery-Otolaryngology, Head and Neck Surgery, Central Adelaide Local Health Network (Basil Hetzel Institute), The Queen Elizabeth Hospital and The University of Adelaide, Adelaide, AS 5011, Australia; mahnaz.ramezanpour@adelaide.edu.au (M.R.); harrison.bolt@uqconnect.edu.au (H.B.); karen.hon@adelaide.edu.au (K.H.); george.bouras@adelaide.edu.au (G.S.B.); alkis.psaltis@adelaide.edu.au (A.J.P.); peterj.wormald@adelaide.edu.au (P.-J.W.); 2 College of Medicine and Public Health, Flinders University, GPO Box 2100, Adelaide, SA 5001, Australia

**Keywords:** chronic rhinosinusitis, ACE2, TMPRSS2, human nasal epithelial cells

## Abstract

**Background**: Viral entry of severe acute respiratory syndrome coronavirus 2 (SARS-CoV2) via the spike protein enables endocytosis into host cells using the ACE2 receptor and TMPRSS2. The frequent upper respiratory tract symptoms of COVID-19 and the localization of the virus to the nasopharynx, the most common site of swabbing, indicate that the sinonasal mucosa may play an important role in SARS-CoV2 infection and viral replication. **Methods:** This paper investigates the presence of ACE2 receptor and TMPRESS2 expression in the primary human nasal epithelial cells (HNECs) from the following: chronic rhinosinusitis without nasal polyps (CRSsNP), CRS with nasal polyps (CRSwNP) and control (non-CRS) patients, and maps the expression changes when exposed to Th1, Th2, Th17-associated cytokines. **Results:** We found that ACE2 and TMPRSS2 expression was higher in control HNECs than CRSwNP HNECs, and that both ACE2 and TMPRSS2 were downregulated further by Th2 cytokines in CRSwNP HNECs. **Conclusions:** This indicates an immune dysregulated state of CRSwNP mucosa, which normally contributes to a chronic inflammatory state, and might support an altered susceptibility to SARS-CoV2 infection and transmission.

## 1. Introduction

The global pandemic following the outbreak of coronavirus disease of 2019 (COVID-19) caused by the severe acute respiratory syndrome coronavirus 2 (SARS-CoV-2) continues to pose a serious health threat. Understanding the pathophysiological cell entry mechanisms of SARS-CoV-2 has been an important area of research in the effort to find a vaccine for COVID-19 [1,2]. The SARS-CoV-2 virus has been shown to exhibit similar cell entry mechanisms to the SARS-CoV virus, which uses the angiotensin-converting enzyme 2 (ACE2) receptor for mammalian cell entry [3,4]. SARS-CoV (SARS) and SARS-CoV-2 (COVID-19) have a similar coronavirus surface spike (S) protein which mediates host cell endocytosis by binding with cellular surface receptors [1]. The spike protein’s S1 subunit is responsible for the receptor binding activity, while the S2 subunit houses the functional elements used for membrane fusion [5,6]. Once the SARS spike protein binds to the ACE2 receptor, it becomes more susceptible to host cell proteolytic digestion [5]. This cleavage is required for efficient complex formation of the S protein with the ACE2 receptor, facilitating virus uptake [6]. A number of proteolytic systems have been outlined in the literature however, transmembrane serine protease 2 (TMPRSS2) has been identified as an independent facilitator of virus entry via the ACE2 receptor [5]. In vitro studies should therefore investigate cellular co-expression of both ACE2 and TMPRSS2 as an important target of SARS-CoV-2 infectability [5,6,7]. ACE2 and TMPRSS2 expression has been demonstrated in many different cells and tissues throughout the body, not just lung and vascular epithelium [8]. This suggests a potential role for ACE2 in viral uptake and replication by multiple cell types and the involvement of multiple organ systems [7], increasing the potential for immune dysregulation.

Indeed, when SARS-CoV-2 infects the host, the normal inflammatory response is key to viral eradication and survival. However, a delayed and dysregulated response can evolve in some cases resulting in a cytokine storm which can be catastrophic to the patient [9]. It was demonstrated that several comorbidities including cancer, diabetes and immunodeficiency, are associated with a higher risk of cytokine storm [10,11,12,13]. Studies showed that type I IFNs (IFNα, IFNβ) and type II IFNs (IFNγ) induced ACE2 expression in nasal epithelial cells. Other studies showed that ACE2 expression was negatively associated with type 2 cytokines, whereas TMPRSS2 expression was positively associated with type 2 cytokines [8,14,15]. Type 2 cytokines are typically increased in allergic diseases and asthma and a recent study has shown that asthma was not an independent risk factor for severe disease or death due to COVID-19 [14]. The incidence of asthma was 23% in patients with CRS compared with the general population which was only 5% [15]. However, no studies have compared the expression of ACE2 and TMPRSS2 in epithelial cells from control patients and patients with chronic rhinosinusitis with (CRSwNP) and without (CRSsNP) nasal polyps.

ACE2 receptor expression is upregulated in a number of conditions including as a protective response to the influenza virus infection [16]. Upregulation of ACE2 receptors as a protective response is likely partially mediated via INFα and INFγ in response to viral invasion [17]. Cytokine induced expression of TMPRSS2 is comparatively less researched, however a number of studies show TMPRSS2 modulation via Th2 cytokines in airway epithelial cells [6,18]. Previous studies into CRS and COVID-19 have shown that sinus epithelial cells and resident cells within the sinus mucosa are able to express cytokines from the Th1, Th2, and Th17 families [19,20]. The level of ACE2 and TMPRSS2 expression in airway epithelial cells and their response to inflammatory cytokines varies in the literature [6,21,22]. This may be in part explained by the low numbers of primary human airway epithelial cell cultures used in in vitro experiments, and the inability to classify the patients by underlying chronic inflammatory disease state [21,23]. A more detailed understanding of ACE2 and TMPRSS2 expression in pathophysiological conditions, in the context of pre-existing morbidities may help to risk-stratify patients and guide treatment [6,22].

The effective application of available laboratory techniques is vital in the management of the SARS-CoV-2 pandemic. Neither the array of initial symptoms associated with COVID-19, nor the delayed ARDS response are pathognomonic for a SARS-CoV-2 infection [24]. PCR based viral swabs remain the gold standard in testing for an early infection [25]. Although the laboratory techniques may alter test-time and sensitivity, the nature of a deep nasopharyngeal swab leaves it open to false negatives due to poor patient compliance, and the potential to miss a cleared viral load [25]. As there are reports of a SARS-CoV-2 ARDS repose with negative nasopharyngeal swabs [26], serum antibody testing can overcome some disadvantages of nasopharyngeal swab testing [25]. However, the delay in becoming seropositive and potential cross reactivity with other common coronaviruses, require antibody testing be employed in a clinically correlated setting. Various techniques used in tandem can help guide severe symptom management [25].

This study investigated ACE2 and TMPRSS2 expression in primary human nasal epithelial cells from control, CRSsNP, and CRSwNP patients. We also evaluated ACE2 and TMPRSS2 expression changes upon challenge with Th1, Th2, and Th17 cytokines families.

## 2. Results

### Clinical Characteristics

HNEC cultures were established from a total of 42 patients [19 women and 23 men and with a mean age of 53 (range, 21–78)]. The patient cohort consisted of 12 controls, 18 CRSsNP and 12 CRSwNP patients. The patient cohort included 23 asthma positive and 19 asthma negative cases. Fifteen patients were challenged with TH1 cytokines (4 controls, 7 CRSsNP and 4 CRSwNP patients). Thirteen were challenged with TH2 and TH17 cytokines (4 controls, 5 CRSsNP and 4 CRSwNP patients).

The symptom scores (SNOT-22 and ADS), GERD status, the LMS and the LK scores for these patients are summarised in Table 1A,B is a summary table for the frequency and percentage of each condition.

**Figure 1:**
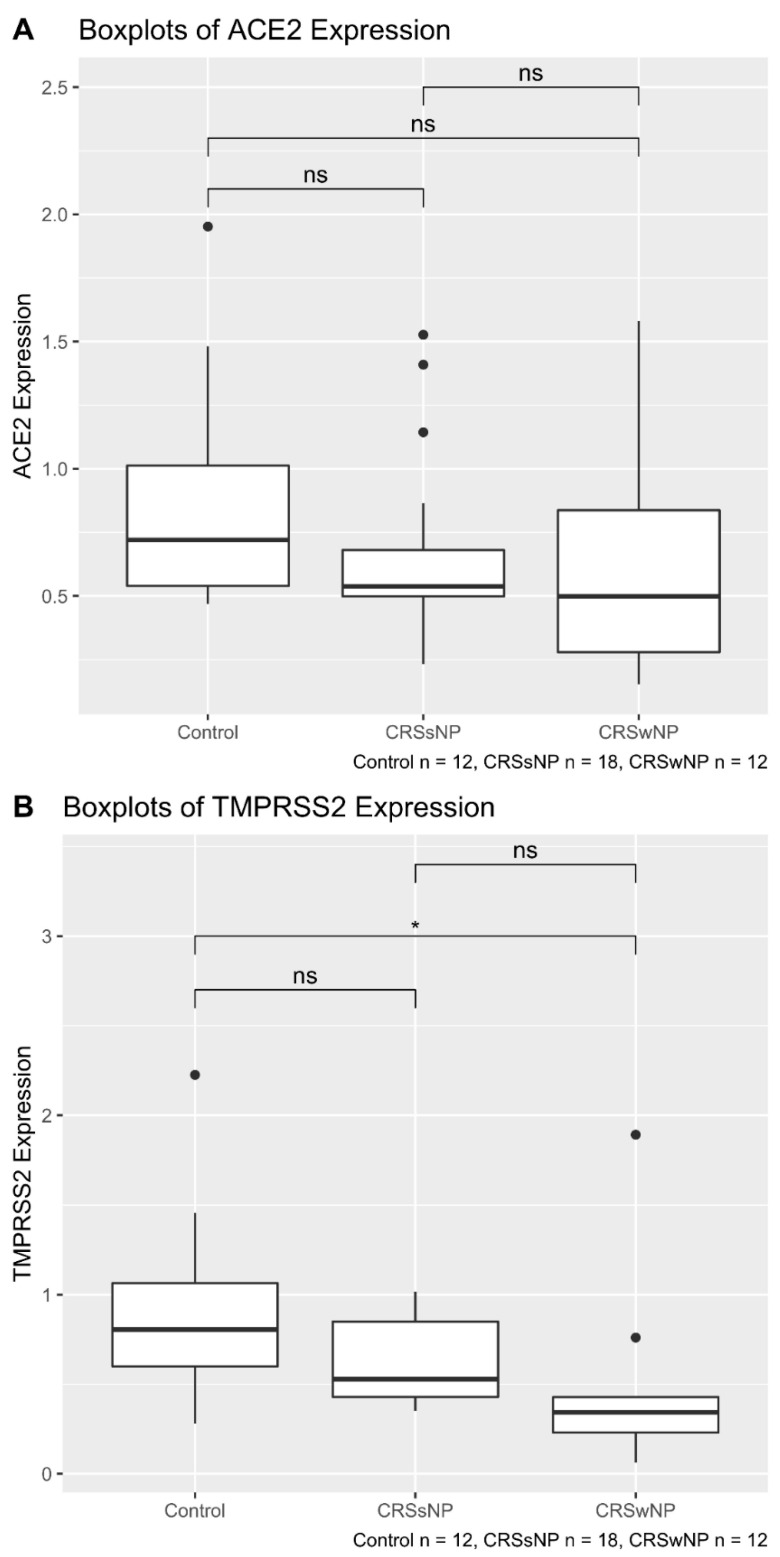
Boxplot showing ACE2 (**A**) and TMPRSS2 (**B**) expression in 3 groups (control, CRSsNP and CRSwNP). The patient cohort consisted of 12 control patients, 18 with CRSsNP and 12 with CRSwNP. (**A**) The mRNA expression of ACE2 does not show any significant difference between groups. (**B**) The TMPRSS2 is significantly different between the groups (1-way ANOVA *p*-value = 0.03). * *p* < 0.05. ns = not significant.

Comparing the mRNA expression of ACE2 and TMPRSS2 between the 3 groups (control, CRSsNP and CRSwNP) showed similar expression of ACE2 across the groups (1-way ANOVA *p*-value = 0.295) (Figure 1A). However, the mRNA expression of TMPRSS2 was significantly different between the groups (1-way ANOVA *p*-value = 0.03). In particular, there was a significantly reduced mRNA expression of TMPRSS2 in CRSwNP patients compared with control patients (Tukey HSD *p*-value = 0.026) (Figure 1B).

**Figure 2:**
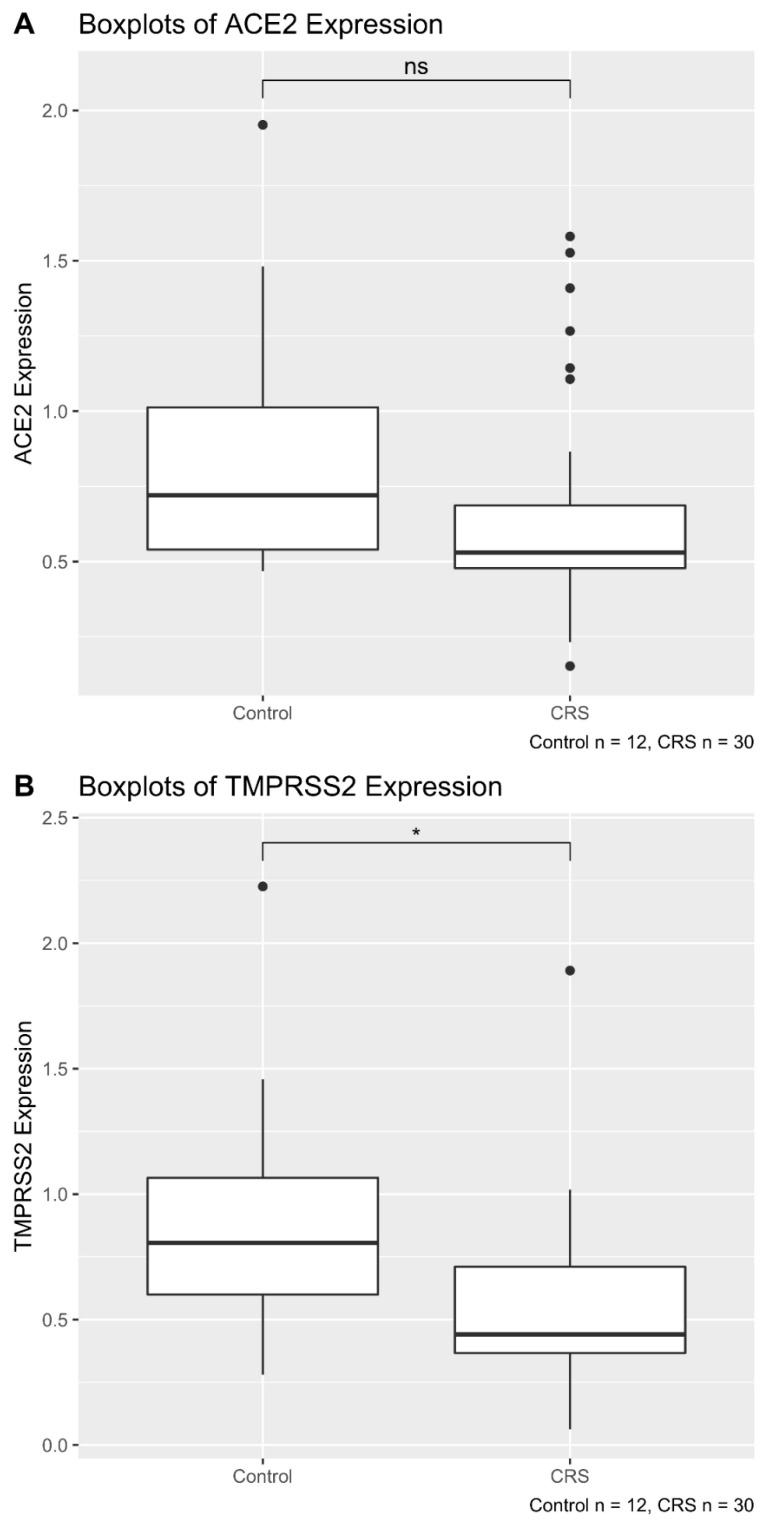
Boxplot showing ACE2 (**A**) and TMPRSS2 (**B**) expression in CRS and control Cases. The patient cohort consisted of 42 patients, including 30 CRS and 12 control cases. (**A**) CRS status is not significantly correlated with ACE2 mRNA expression (Student *t*-test *p*-value = 0.12). (**B**) CRS is significantly correlated with a reduced TMPRSS2 expression (Student *t*-test *p*-value = 0.014). * *p* < 0.05. ns = not significant.

When we grouped the patients in controls vs CRS patients (including CRSwNP and CRSsNP), the analysis showed that the CRS status was not significantly correlated with ACE2 mRNA expression (Student’s *t*-test *p*-value = 0.12) (Figure 2A). However, CRS was significantly correlated with reduced TMPRSS2 expression (Student *t*-test *p*-value = 0.014) (Figure 2B).

The disease severity scores, such as the Visual Analogue Scale (VAS) and Sinonasal Outcome Test-22 (SNOT-22) can be used in CRS to evaluate the burden of disease [23]. Therefore, we looked at the relationship between demographic factors, disease severity metrics and ACE2 or TMPRSS2 expression. Age, gender, GERD, SNOT-22, the Lund-Mackay, the disease-specific 5-question-based Adelaide Severity score (ADS) and the Lund-Kennedy scores were not significantly correlated with ACE2 or TMPRSS2 expression (all *p*-values > 0.05). The patients that received oral steroids were divided into 4 roughly equal groups depending on whether and when they received those treatments (never, 0–3 months, 4–6 months, and 6+ months). There was no significance between steroid timing and ACE2 expression (*p* = 0.375 1-way ANOVA) or TMPRSS2 expression (*p* = 0.809 1-way ANOVA) (Figure S1A,B). Moreover, the patients that had steroids nasal wash were divided into two groups “currently” vs “everything else (not currently)”. There was no significant difference in expression of ACE2 (Student *t*-test *p* = 0.51) or TMPRSS2 (Student *t*-test *p* = 0.76) between “currently” vs. “not currently” usage of steroids nasal wash (Supplementary Figure S1C,D).

**Figure 3:**
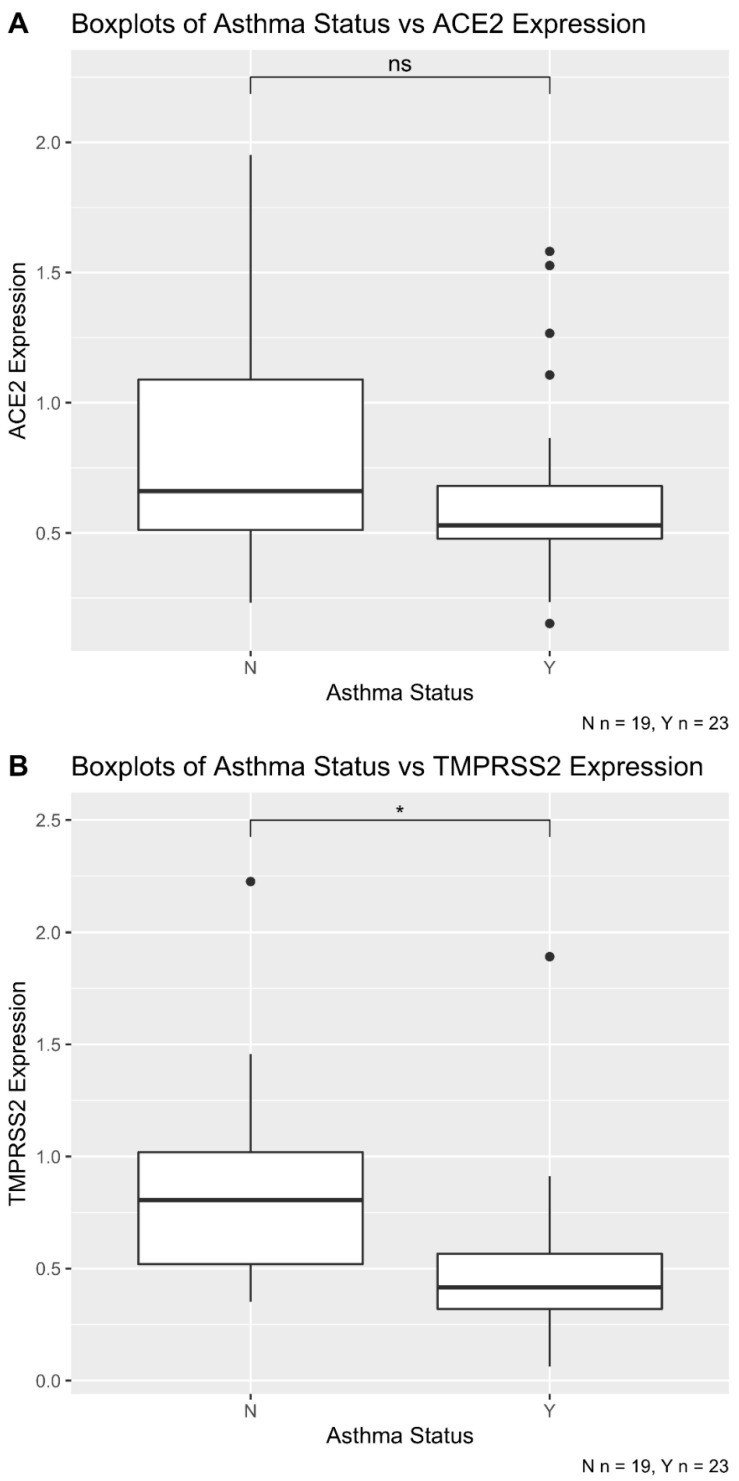
Boxplot of asthma status versus ACE2 (**A**) and TMPRSS2 (**B**) expression. The patient cohort consisted of 42 patients including 23 asthma and 19 non-asthma cases. (**A**) Asthma is not significantly correlated with ACE2 expression (Student *t*-test *p*-value = 0.18). (**B**) Asthma patients show significant reduction of TMPRSS2 mRNA expression compared with non-asthma patients (Student *t*-test *p*-value = 0.01). * *p* < 0.05. ns = not significant.

Asthma was not significantly correlated with ACE2 expression (Student *t*-test *p*-value = 0.18) (Figure 3A). However, asthma positive patients showed a significant reduction in TMPRSS2 mRNA expression compared with asthma negative patients (Student *t*-test *p*-value = 0.01) (Figure 3B).

**Figure 4:**
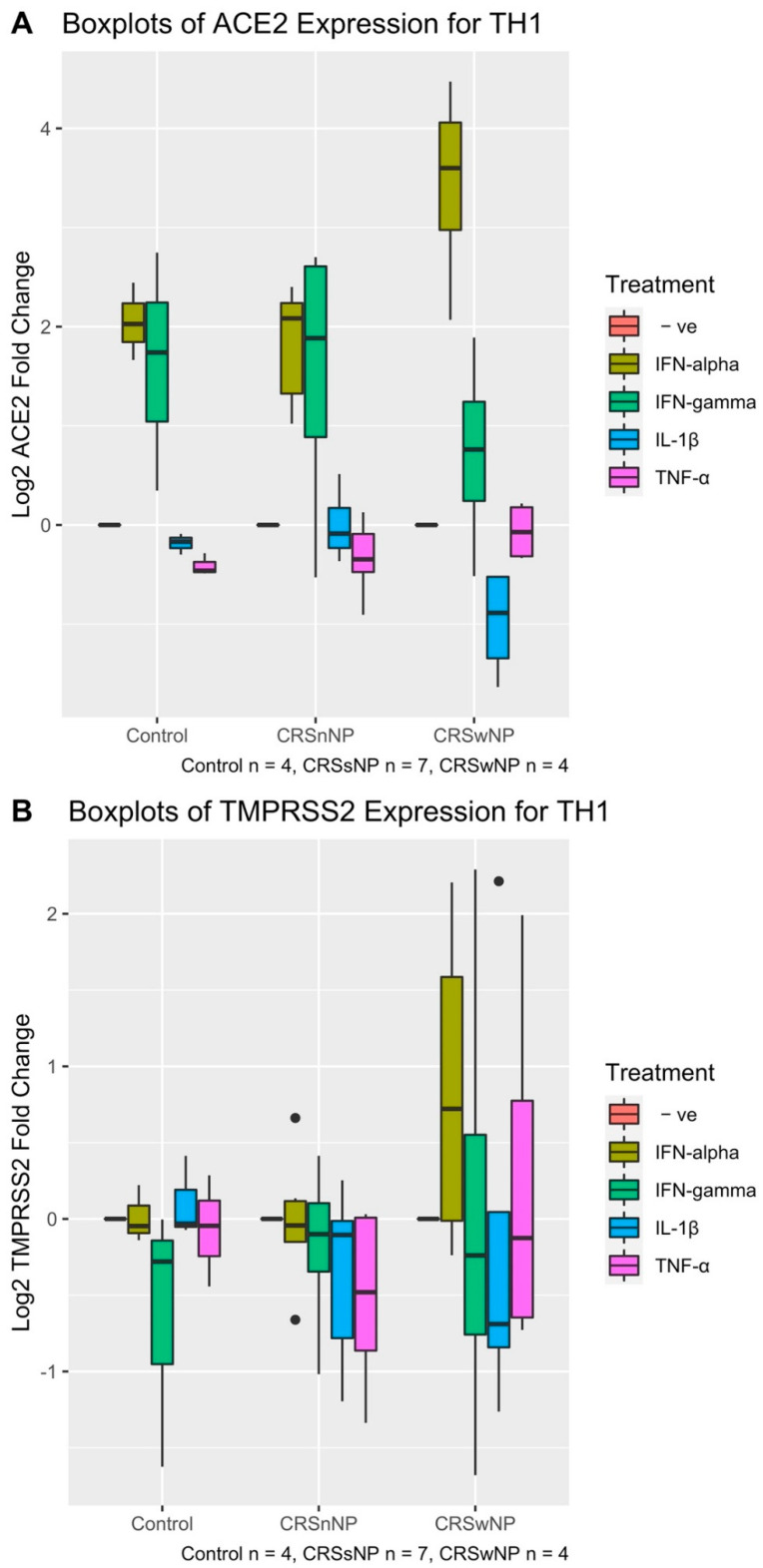
Boxplot log of ACE2 (**A**) and TMPRSS2 (**B**) expression of human nasal epithelial cells treated with IFN-α, IFN-γ, IL-1β, TNF-α for 24 h. The patient cohort consisted of 4 patients with CRSwNP, 7 with CRSsNP and 4 control cases. (**A**) IFN-α and IFN-γ show significantly higher ACE2 mRNA expression compared with untreated cells and the other treatment groups (Tukey HSD *p*-values all < 0.001). (**B**) There are no significant effects on TMPRESS2 expression for HNECs treated with IFN-α, IFN-γ, IL-1β and TNF-α within each of the control, CRSsNP and CRSwNP groups (2-way ANOVA *p*-value = 0.162).

HNECs were treated with IFN-α, IFN-γ, IL-1β and TNF-α for 24 h. Differences in the ACE2 and TMPRSS2 expression between these treatment groups were initially analysed using a 2-way ANOVA to control for the CRS group. For ACE2, there was no significant difference in expression between the 3 groups (control vs CRSsNP vs. CRSwNP) (2-way ANOVA *p*-value = 0.998). Therefore, CRS group was removed from the analysis and a 1-way ANOVA between ACE2 expression and treatment was conducted. The treatment type was statistically significant (1-way ANOVA *p*-value = 5.68 × 10^−15^). In particular, IFN-α and IFN-γ-treated cells showed significantly higher ACE2 mRNA expression compared with untreated cells and the other treatment groups (Tukey HSD *p*-values all < 0.001 for both treatments compared with all other treatment groups). TNF-α and IL-1β treatment groups did not show significantly different ACE2 expression from untreated cells and between each other (Figure 4A). There were no significant effects on TMPRSS2 expression for HNECs treated with IFN-α, IFN-γ, IL-1β and TNF-α within each of the control, CRSsNP and CRSwNP groups (2-way ANOVA *p*-value = 0.162). (Figure 4B). The treatment type was also not statistically significant (2-way ANOVA *p*-value = 0.462) (Figure 4B).

**Figure 5:**
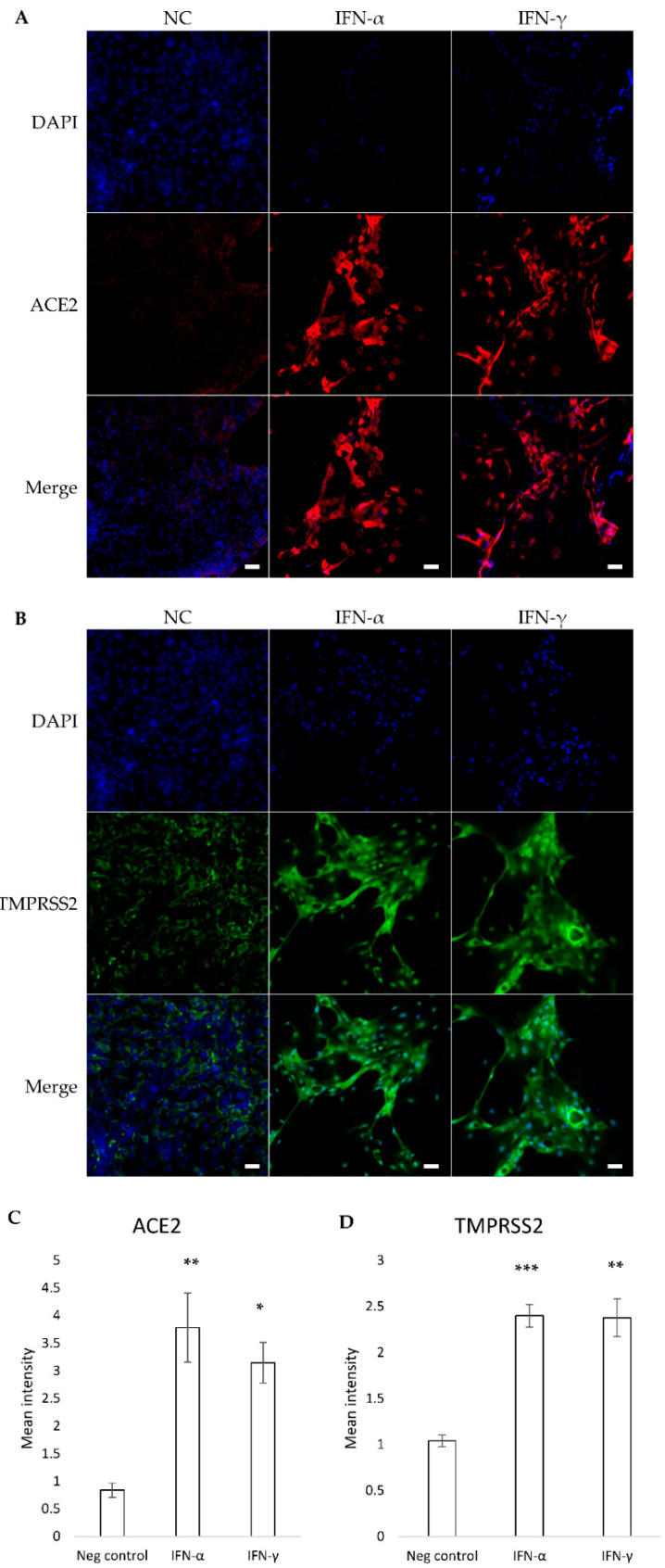
ACE2 protein expression (**A**) and TMPRSS2 protein expression (**B**) in human nasal epithelial cells was determined using immunofluorescence staining for ACE2 (red), TMPRSS2 (green) and nuclei (blue) in the absence (no treatment control-NC) and presence of IFN-α or IFN-γ. The white bar is 50 µm and 20 × objective. ACE2 (**C**) and TMPRSS2 (**D**) fluorescence intensity of HNEC cultures (harvested form inferior turbinates from CRS) with IFN-α and IFN-γ treatments. The values are shown as means ± SEM, * *p* < 0.05, ** *p* < 0.01, *** *p* < 0.001, n = 6.

Given that IL-1β and TNF-α did not change the expression of ACE2 at the mRNA level, we continued the immunofluorescence staining of HNEC cultures (harvested from inferior turbinates from CRS) with IFN-α and IFN-γ treatments only. The immunofluorescence staining showed that IFN-α and IFN-γ increased the protein expression of ACE2 (Figure 5A) and TMPRSS2 (Figure 5B). Moreover, we examined ACE2 and TMPRSS2 protein expression by using immunofluorescence (Figure 5C,D). The intensity of ACE2 in HNEC treated with IFN-α (Tukey HSD *p* = 0.00478) and IFN-γ (Tukey HSD *p* = 0.015) significantly increased in comparison with untreated cells (Figure 5C). The TMPRSS2 intensity was also increased with IFN-α (Tukey HSD *p* = 0.0009) and IFN-γ (Tukey HSD *p* = 0.001) compared with untreated cells (Figure 5D).

**Figure 6:**
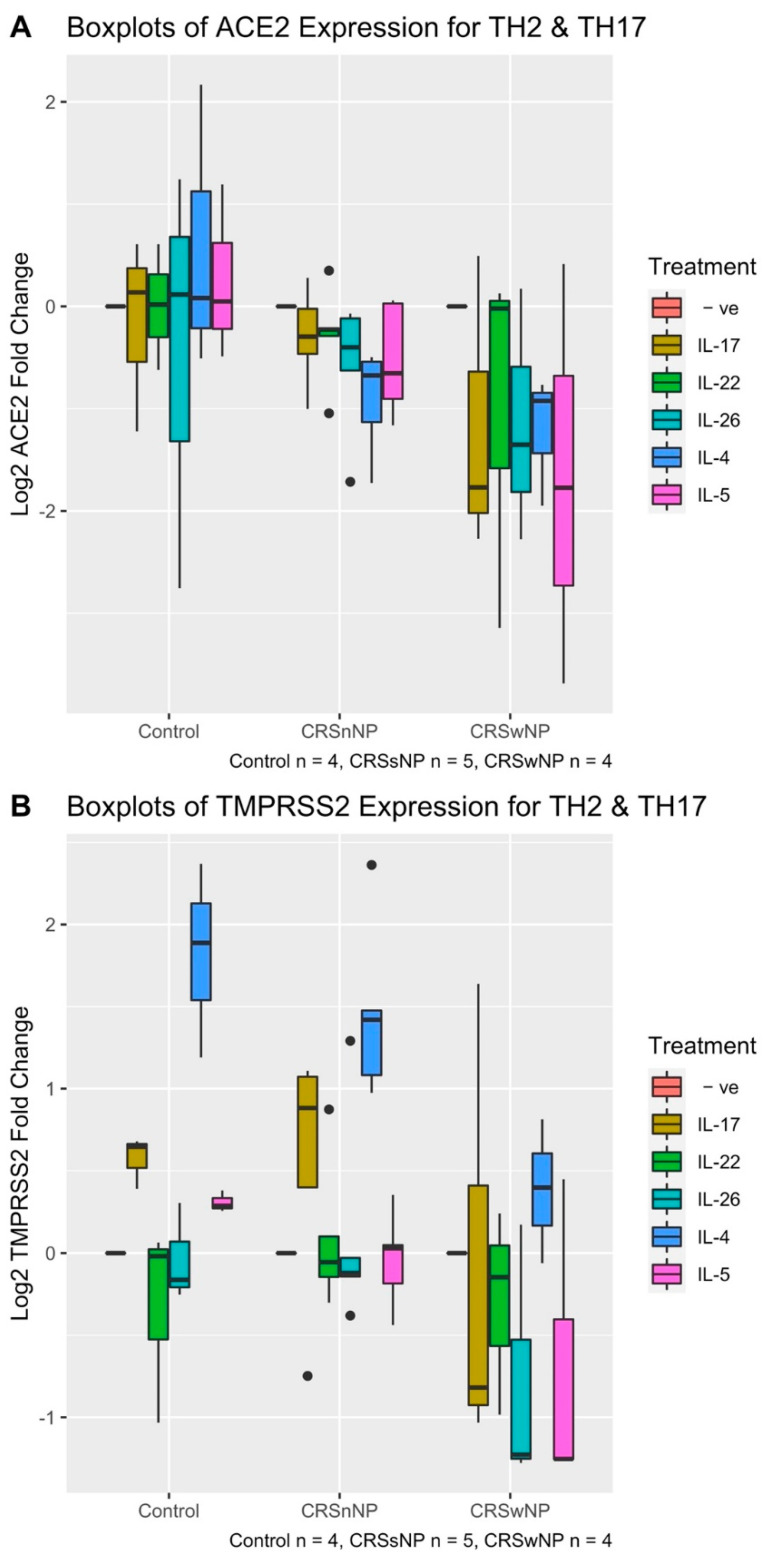
Boxplot log of ACE2 (**A**) and TMPRSS2 (**B**) expression of human nasal epithelial cells after treating with TH2 and TH17 families for 24 h. The patient cohort consisted of 4 patients with CRSwNP, 5 with CRSsNP and 4 control cases. (**A**) ACE2 mRNA expression differed significantly between CRSwNP group (1-way ANOVA *p*-value = 0.00463) though not between the TH2 and TH17 cytokines (2-way ANOVA *p*-value = 0.557). (**B**) TMPRSSS expression differed between CRS groups (2-way ANOVA *p*-value = 0.0378) and treatment groups (2-way ANOVA *p*-value = 2.05 × 10^−7^).

We next challenged HNECs with Th2 (IL-4, IL-5) or Th17 (IL-17, IL-22, IL-26) cytokines for 24 h followed by evaluating the expression of ACE2 and TMPRSS2. Differences in the ACE2 expression between these treatment groups and between the CRS condition groups were first analysed using a 2-way ANOVA. ACE2 mRNA expression was not different between the various Th2 and Th17 cytokine treatment groups (2-way ANOVA *p*-value = 0.557). Therefore, the treatment group was removed as a variable in the ANOVA analysis. After conducting a 1-way ANOVA analysis between ACE2 expression and CRS status, there was a significant difference in ACE2 expression between CRS and control groups (1-way ANOVA *p*-value = 0.0046). On average, CRSwNP patients had a 2.10-fold reduction in expression of ACE2 compared with control patients after stimulation with Th2 and Th17 cytokines (HSD *p*-value 0.001). In contrast, ACE2 expression did not change significantly between CRSsNP and control patients (HSD *p*-value = 0.2172) after challenge with Th2 and Th17 cytokines (Figure 6A).

When the same analysis was repeated for TMPRSS2, 2-way ANOVA revealed that TMPRSS2 expression was significantly different between CRS and control groups controlling for treatment type (2-way ANOVA *p*-value = 0.0378), as is consistent with the finding evident in Figure 1B, which shows a reduction in TMPRSS2 expression for CRSwNP patients as compared with control group patients. This can be seen in the overall structure of Figure 6B: the CRSwNP boxplots, coloured for each cytokine, show lower levels for expression than their cognate in the CRSsNP and control groups. On average, HNECs derived from CRSwNP patients showed an average 1.55-fold TMPRSS2 reduction in expression compared with control patients across Th2 and Th17 cytokine treatments (HSD *p*-value = 0.04) and a 1.52-fold reduction in expression compared with CRSsNP patients (HSD *p*-value = 0.027) (Figure 6B), when controlling for the cytokine treatment group.

Two-way ANOVA analysis also showed there was a significant difference between the cytokines treatment groups (2-way ANOVA *p*-value = 2.05 × 10^−7^) when controlling for CRS group. In particular, IL-4 was significantly different in modulating TMPRSS2 expression in HNECs cells compared with all other cytokine treatments, controlling for CRS and control patient groups (HSD *p*-values ranged from 3.6 × 10^−5^ to 0.027, testing the difference between IL-4 and each of the other cytokine treatment and negative control). On average, IL-4 treated HNECs showed a 2.40-fold TMPRSS2 expression increase compared with the negative control (untreated cells). Additionally, the 2-way ANOVA interaction term between CRS group and cytokine treatment group was not significant (2-way ANOVA interaction term *p*-value = 0.18). This suggests that the upregulation of TMPRSS2 by IL-4 is independent of the finding that TMPRSS2 shows reduced expression in CRSwNP patients (Figure 1B). This is demonstrated by Figure 6B: the blue boxplots, indicating IL-4 TMPRSS2 expression for each CRS group, show significantly higher expression compared with the other coloured boxplots (for each other cytokines and the negative control) within each CRS group. Further, when comparing the blue boxplots between CRS groups, it is clear that the expression is lowest in CRSwNP group. Other cytokines, including IL-17, IL-22, IL-26, IL-5 did not induce any significant change in TMPRSS2 expression (HSD *p*-value > 0.5) compared with untreated cells (Figure 6B) when controlling for CRS group.

## 3. Discussion

This study shows that ACE2 and TMPRSS2 expression in human sinonasal epithelial cells differs depending on the host’s sinus mucosa pathological state. While the pathophysiology of chronic rhinosinusitis remains elusive, research shows that there is an imbalance in the normal microbiome as well as underlying innate immune dysregulation [27]. This dysregulated state supports the findings that ACE2 expression is lower in CRS patients. Sinonasal epithelium shows high rates of double positive ACE2 and TMPRSS2 expressing cells [23] which, given that nasopharyngeal swabs have become the gold standard for COVID-19 testing [28], highlights the importance of the sinonasal mucosa as both an portal for SARS-CoV-2 infection and replication in human hosts. In addition, studies showed other routes, involving neurotropic and neurovirulent pathways may play roles in SARS-CoV2 invasion. SARS-CoV2 is able to infect the brainstem via nerve terminals in the orofacial mucosa, eyes, and olfactory neuroepithelium which act as entry points to the central nervous system [29,30,31].

High levels of ACE2 expression are important in identifying the role of the sinonasal mucosa in COVID-19 infections, combined with the concurrent cytokine modulated TMPRSS2 expression supports the hypothesis that SARS-CoV-2 can not only bind to the many receptors in the nose, but also undergo proteolytic cleavage and endocytosis in the upper airway [23]. Severity of symptoms differs greatly between patients, and the link between ACE2 and TMPRSS2 expression with disease burden is poorly understood. Due to the significant heterogeneity in case presentations, gaining a better understanding of ACE2 and TMPRSS2 immune pathways activated in different organ tissues on the background of varied pre-morbid states is a key component of understanding SARS-CoV2 pathophysiology and establishing ACE2-related therapies in the setting of severe infections. The link between worse outcomes in patients with pre-existing respiratory disease, such as asthma, chronic inflammatory conditions, or immunosuppressed status and SARS-Cov2 infection severity is difficult to attribute purely to either an altered inflammatory reaction in response to the pathogen, or simply due to a poor pre-infection baseline. With these reasons in mind, this paper explores the Th1 and Th2 cytokine expression in upper airway mucosal tissue in vitro from hosts with varied disease backgrounds [4,32].

Our results showed no significant difference between ACE2 expressions in asthma patients compared with those who do not have asthma; however, there was a difference in TMPRSS2 expression in asthma patients vs controls. Clinical risk factors for severe COVID-19 cases are poorly classified due to the ever-evolving pool of patient data obtained during the 2020 pandemic. Regardless, age, end-stage organ failure, viral infections, cardiovascular disease, sepsis, obesity, severe immunosuppression, cancer, and poorly controlled or end-stage respiratory diseases are identified as risk factors increasing the morbidity and mortality from a SARS-CoV-2 infection [10,11,12,13]. The literature presents mixed findings regarding the mortality risk of COVID-19 patients with prior diagnosis of asthma and upper airway diseases [33]. This may be in part due to the heterogeneous innate immune patterns found in these patients. The other reason that why asthma is listed as a risk factor for COVID-19 morbidity is because the acute respiratory syndrome coronavirus 2 (SARS-CoV-2) triggers asthma exacerbations. As resent studies showed that asthma patients are overrepresented among the adult patients who have been admitted to hospital with coronavirus disease 2019 [34]. Further investigating the expression of ACE2 and TMPRSS2 expression in sinonasal epithelial cells from diseased and healthy patients at baseline may help to further subclassify patients with broad clinical diagnosis and better target risk management and treatment options.

The use of primary cells sourced from multiple different patients and categorised according to chronic sinus disease diagnosis allows a deeper understanding into some of the conflicting results published regarding ACE2 and TMPRSS2 expression in response to different interferons and interleukins [5,15,21,35]. The pre-existing innate immune state of the host cell plays a significant role in the response to cytokines. Type 1 and Type 2 interferons increased ACE2 expression in each patient group. This is in keeping with the expected changes based on the ACE2 upregulation in response to influenza and other viruses [35]. Apart from IL-4 cytokine, IL-5, IL-17, IL-22, and IL-26 cytokines did not modulate ACE2 and TMPRSS2 expression in control patients, however strongly down regulated expression of both proteins in CRSwNP cells. This may suggest there are more long-term epigenetic changes responsible for the expression differences between non-CRS and CRS patients [21]. The Th2 cytokine pathway has been implicated in the modulation of ACE2 expression in the literature, however the potential for epigenetic changes influencing its expression are poorly understood [15,18]. There is an evolving body of research to suggest a chronic switch to a Th2 pathway in CRSwNP patients [36]. Understanding the innate immune dysregulation contributing to CRS and the role of ACE2 in the innate response to viral infection is in its infancy. Although the clinical significance is difficult to elucidate from this information, the strong down regulation of both ACE2 and TMPRSS2 in CRSwNP epithelial cells in response to exogenous Th2 cytokines presents an area of further research.

Our findings are indicative of differences in expression between cell types coming from those different patient phenotypes. To investigate the potential role of differences in pathophysiology, a further study would have to be carried out comparing gene expression changes in HNECs between sampling sites (nasal polyps and inferior turbinates). Certain limitations must be considered for the present study. The ACE2 and TMPRSS2 expression levels were assessed with immunofluorescence. Confirming these results with other assays such as a Western blot would be beneficial.

In conclusion, the response to Th1, Th2 and Th17 cytokines is different between control and CRSwNP. Further research into the clinical significance of different ACE2 and TMPRSS2 co-expression in terms of disease severity is required. Gaining a better understanding into the role of ACE2 in the non-RAAS associated response to viral infection in airway tissues is important to chronic airway disease and acute SARS-CoV-2 infections.

## 4. Materials and Methods

### 4.1. Patients

Our study was approved for five years (from 29 January 2018–29 January 2023) by The Central Adelaide Local Health Network Human Research Ethics Committee (CALHN HREC). Informed consent was obtained from all subjects involved in the study (HREC/18/CALHN/69). Exclusions included active smoking and age less than 18 years (none of the specimens were from COVID-19 patients). Chronic rhinosinusitis (CRS) patients fulfilled the diagnostic criteria for CRS according to the recent position papers by the American Academy of Otolaryngology and Head and Neck Surgery and the European Position Statement (EPOS) [37] on CRS. Patients with CRS were further sub-classified according the absence (CRSsNP) or presence (CRSwNP) of nasal polyps as defined by the EPOS guidelines [38]. Clinical data from the patients were collected prospectively including age, gender, gastro-oesophageal reflux disease (GERD), number of operations and history of asthma. Disease severity was measured based on completion of the preoperative patient-reported 22-item Sino-Nasal Outcome Test (SNOT-22) questionnaire [39], the disease-specific 5-question-based Adelaide Severity score (ADS) [40] and the objective computed tomography (CT)- scan measure of Lund-Mackay score (LMS) [41].

### 4.2. Primary Human Nasal Epithelial Cell Culture

Primary human nasal epithelial cells (HNECs) were harvested from the inferior turbinates by gentle brushing from patients who were undergoing endoscopic skull base surgery or septoplasty and had no clinical or radiologic evidence of sinus disease (control) and from patients with CRSsNP. HNECs from CRSwNP were harvested by gentle brushing of nasal polyps under endoscopic guidance. Nasal brushings were suspended in Nasal Epithelial Growth Media (STEMCELL Technologies Australia Pty. Ltd., Tullamarine, VIC, Australia). Extracted cells were then depleted of monocytes using anti-CD68 (Dako, Glostrup, Denmark) coated culture dishes. HNECs were expanded in routine cell culture conditions of 37 °C humidified air with 5% CO2 in collagen-coated flasks (Thermo Scientific, Walthman, MA, USA). HNECs were used at passage 1 or 2 [42].

### 4.3. Th1, Th2, and Th17 Cytokines Exposure

HNECs were seeded onto collage- coated 6-well dishes at 0.6 × 106 cells/well for 24 h prior to the experiment. Cytokines were added to the HNECs at the following final concentrations for 24 h: recombinant human Interferon-γ (500 ng/mL, Sigma, Saint Louis, MI, USA), interferon-α (500 ng/mL, Sigma, Saint Louis, MI, USA), Tumour Necrosis Factor-α (500 ng/mL, Sigma, Saint Louis, MI, USA), IL-1β (500 ng/mL, Sigma, Saint Louis, MI, USA), IL-4 (50 ng/mL, Gibco, Life Technology, Waltham, MA, USA), IL-5 (50 ng/mL, Gibco, Life Technology, USA), IL-17A (50 ng/mL, Gibco, Life Technology, USA), recombinant human IL-22 (50 ng/mL, Sigma, Saint Louis, MI, USA), and recombinant human IL-26 (50 ng/mL, Abnova Taiwan Corp, Taiwan) [43].

### 4.4. RNA Extraction, Reverse Transcription and qPCR

HNECs were seeded onto collagen-coated 6-well dishes at 0.8 × 10^6^ cells/well for 24 h prior the RNA extraction. Total RNA was extracted from HNECs using the Qiagen RNeasy Mini kit (Qiagen GmbH, Hilden, Germany) according to the manufacturer’s instructions followed by DNase treatment with RNase-Free DNase set (Qiagen). Extracted RNA was assessed for quality using the Experion RNA StdSens analysis kit (Bio-Rad Laboratories, Hercules, CA, USA) and total quantification using the Nanodrop 1000 spectrophotometer (Thermo Fisher Scientific, Franklin, MA, USA). RNA was reverse transcribed into cDNA using Quantitect Reverse Transcription kit (Qiagen, Hilden, Germany) with a MyCycler Thermal Cycler (BioRad Laboratories Inc., Gladesville, Australia). The resulting cDNA was subjected to qPCR with TaqMan primer/probe sets for each target gene, TaqMan Universal Master Mix II (Thermo Fisher Scientific, Scoresby, Australia) and nuclease-free water. The average threshold cycle (Ct) was determined from three independent experiments and the level of gene expression relative to Glyceraldehyde 3-phosphate dehydrogenase (GAPDH) was determined with the comparative CT method [44]. TaqMan Gene Assays used for gene expression analysis were: Hs00987595_m1 (ACE2), Hs01122322_m1 (TMPRSS2), and Hs02758991 (GAPDH).

### 4.5. Immunofluorescence

The cells were fixed with 2.5% formalin in PBS for 10 minutes at room temperature (RT) followed by washing with PBS twice. Fixed samples were blocked for 1 h with Serum-Free Protein Block (SFB; Dako, Glostrup, Denmark). Rabbit Anti-ACE2 Polyclonal Antibody (1:100, Invitrogen, Carlsbad, CA, USA) and rabbit Anti-TMPRSS2 antibody (1:100, Abcam, Cambridge, MA, USA) were added overnight at 4 °C. Excess primary antibody was removed, and 2 μg/mL anti-mouse Alexa-Fluor 488 conjugated secondary antibody (Jackson ImmunoResearch Labs Inc., West Grove, PA, USA) was added and incubated for 1 h at RT. The samples were rinsed in TBST, and after the third wash, 200 ng/mL of 4′, 6-diamidino-2-phenylindole (DAPI; Sigma Aldrich, St. Louis, MO, USA) was added to resolve nuclei. Samples were visualized by using a LSM700 confocal laser scanning microscope (Zeiss Microscopy, Germany). Processing was performed using ZEN Imaging Software (Carl Zeiss AG, Oberkochen, Germany). ACE2 and TMPRSS2 fluorescence intensity were quantified and normalized to the DAPI intensity. Results are expressed as relative value of mean arbitrary fluorescence units, provided by the ZEN imaging software.

### 4.6. Statistical Analysis

All statistical analysis was performed in R v3.3.3. For the q-PCR analysis, relative mRNA gene expression was calculated for each isolate and treatment type against a negative control using the 2−ΔΔCT method [44]. All relative mRNA gene expression scores were log base 2 transformed to remove skew in subsequent analyses. The analysis of the impact of various clinical characteristics on ACE2 and TMPRSS2 expression was performed using three methods. Where the predictor variable was a categorical variable with greater than 2 categories, the 1-way ANOVA method was used. Where the predictor variable was a categorical variable with exactly 2 categories, we used the Student *t*-test method. If the categorical variable was numeric, simple linear regression was used. All methods had a significance level of 0.05. The significance of group differences in gene expression between the 3 different CRS groups was determined using the Tukey Honestly Significant Difference (‘HSDs’) method. Where observations were missing for certain demographic characteristics, those samples were omitted from the analysis. The analysis of the impact of different interferon treatments and CRS groups on ACE2 and TMPRSS2 expression was conducted using the 2-way and 1-way ANOVA methods as indicated, with a significance level of 0.05. The significance of differences between individual interferon treatments and CRS groups was determined using the Tukey HSDs method. Visualisations were produced with the R package ‘ggplot2’ v3.3.2 [45].

## Figures and Tables

**Table 1:** Demographics table of HNEC donors (**A**) summary table of frequency and percentage of each condition (**B**) CRSsNP = chronic rhinosinusitis without nasal polyps; CRSwNP = chronic rhinosinusitis with nasal polyps; GOERD = gastro-oesophageal reflux disease; SNOT-22 = Sino-Nasal Outcome Test 22; LMS = Lund-Mackay score; LK= Lund-Kennedy score; ADS = Adelaide Severity score; NA = not available.

**A**												
	**Sex**	**Age**	**Condition**	**Asthma Status**	**GERD**	**SNOT22**	**LMS**	**LKS**	**ADS**	**TH1/TH2/TH17 Challenge**	**Oral Steroids**	**Nasal Wash or Rinse**
1	M	61	Control	N	N	NA	NA	NA	NA			
2	M	75	Control	N	N	NA	NA	NA	NA			
3	F	76	Control	N	Y	NA	NA	NA	NA			
4	F	21	Control	N	N	NA	NA	NA	NA			
5	M	63	Control	N	N	NA	NA	NA	NA			
6	F	60	Control	N	N	NA	NA	NA	NA			
7	M	38	Control	N	Y	NA	NA	NA	NA			
8	M	68	Control	N	N	NA	NA	NA	NA			
9	F	33	Control	Y	N	NA	NA	NA	NA	Th1/TH2/TH17		
10	F	68	Control	N	N	NA	NA	NA	NA	Th1/TH2/TH17		
11	M	73	Control	Y	N	NA	NA	NA	NA	Th1/TH2/TH17		
12	F	58	Control	Y	Y	NA	NA	NA	NA	Th1/TH2/TH17		
13	M	60	CRSsNP	N	Y	39	6	4	44		>12 months ago	10 days ago
14	M	71	CRSsNP	Y	Y	45	11	10	33.5		6 weeks ago	currently
15	M	78	CRSsNP	Y	Y	41	8	12	44.9		never	currently
16	M	54	CRSsNP	N	N	25	8	7	NA		4 months ago	currently
17	F	69	CRSsNP	Y	N	56	9	6	20.2		>2 weeks ago	currently
18	F	51	CRSsNP	Y	N	27	0	7	9		never	currently
19	M	34	CRSsNP	Y	N	14	4	10	6		>12 months ago	currently
20	M	68	CRSsNP	N	N	33	8	12	47.1		5 months ago	4 months ago
21	F	27	CRSsNP	Y	Y	57	1	4	NA	Th1/TH2/TH17	5 months ago	currently
22	M	50	CRSsNP	N	N	4	2	4	NA	TH1 only	3 weeks ago	2 months ago
23	M	64	CRSsNP	Y	N	53	13	4	15	TH1 only	>6 months ago	currently
24	F	53	CRSsNP	N	N	51	12	12	NA	TH1 only	8 months ago	3 months ago
25	M	69	CRSsNP	N	Y	46	4	4	NA	TH1 only	never	currently
26	F	52	CRSsNP	Y	N	50	4	9	NA	Th1/TH2/TH17	2 months ago	currently
27	F	64	CRSsNP	N	N	69	10	8	NA	Th1/TH2/TH17	never	currently
28	F	24	CRSsNP	Y	N	50	6	6	NA	TH2/TH17 only	3 months ago	4 weeks ago
29	M	26	CRSsNP	Y	N	NA	5	4	NA	TH2/TH17 only	never	currently
30	M	54	CRSsNP	N	N	18	1	5	NA		>6 months ago	>6 months ago
31	F	36	CRSwNP	Y	N	43	NA	NA	38		>12 months ago	currently
32	F	51	CRSwNP	Y	N	45	22	NA	44.6		6 months ago	currently
33	M	42	CRSwNP	Y	Y	7		NA	14.6		4 months ago	currently
34	F	57	CRSwNP	Y	N	75	19	14	46.1		7 months ago	currently
35	F	28	CRSwNP	Y	N	63	23		58.9		2 months ago	currently
36	F	61	CRSwNP	Y	N	22	21	16	20		>12 months ago	currently
37	M	36	CRSwNP	Y	N	45	18	10	40		10 months ago	currently
38	F	51	CRSwNP	Y	N	57	16	15	31.5		>12 months ago	>12 months ago
39	M	74	CRSwNP	N	N	NA	24	5	NA	Th1/TH2/TH17	never	NA
40	M	69	CRSwNP	Y	N	7	6	6	NA	Th1/TH2/TH17	>6 months ago	3 weeks ago
41	M	21	CRSwNP	N	N	68	21	12	NA	Th1/TH2/TH17	never	3 months ago
42	M	75	CRSwNP	Y	N	31	8	13	NA	Th1/TH2/TH17	never	currently
**B**												
CRS Condition				Control						CRSsNP	CRSwNP	Total
Frequency				12						18	12	42
Percentage				28.6%						42.9%	28.6%	100.0%
				Sex						F	M	Total
				Frequency						19	23	42
				Percentage						45.2%	54.8%	100.0%
				Asthma						N	Y	Total
				Frequency						19	23	42
				Percentage						45.2%	54.8%	100.0%
				GERD						N	Y	Total
				Frequency						33	9	42
				Percentage						78.6%	21.4%	100.0%

## Data Availability

Data is contained within the article or supplementary material. The Data presented in this study are available in [Cytokine-Induced Modulation of SARS-CoV2 Receptor Expression in Primary Human Nasal Epithelial Cells].

## Supplementary Materials

The following are available online at https://www.mdpi.com/article/10.3390/pathogens10070848/s1, Figure S1. ACE2 protein expression (A) and TMPRSS2 protein expression (B) in CRS patients that taken up oral steroids. The oral steroid divided into 4 roughly equal groups (never, 0–3 months, 4–6 months, and 6+ months). ACE2 protein expression (C) and TMPRSS2 protein expression (D) in CRS patients that used steroids nasal wash. The patients that had steroids nasal wash divided into two groups “currently” vs. “not currently”. The values are shown as means ± SEM, 1way ANOVA, n = 30.
