# Supplementary figures and images for: Cytokine-Induced Modulation of SARS-CoV2 Receptor Expression in Primary Human Nasal Epithelial Cells
Source: Pathogens. 2021 Jul 5;10(7):848. doi: 10.3390/pathogens10070848 (PMC8308731; doi:10.3390/pathogens10070848)

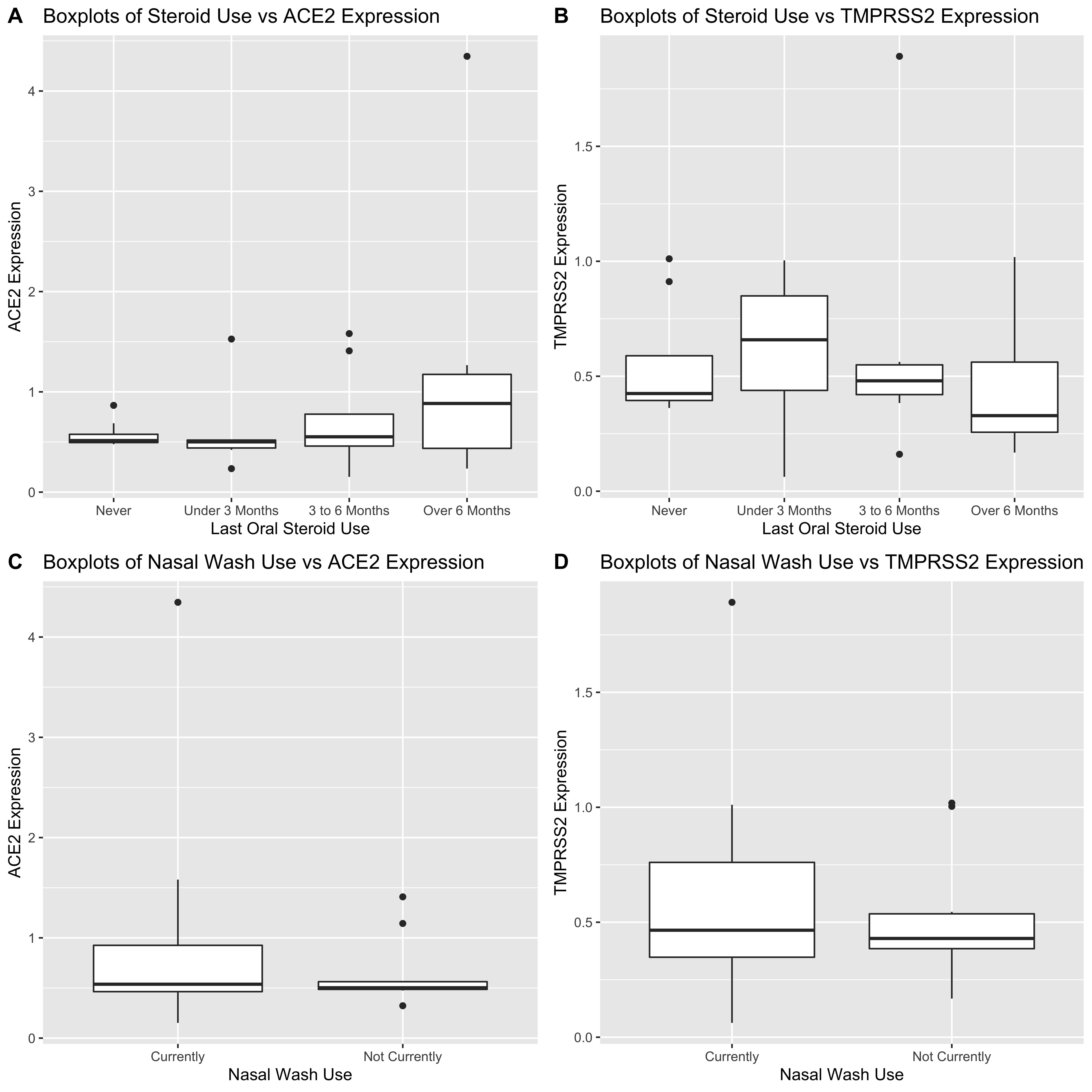

Supplement: Supplementary file 1 [file pathogens-10-00848-s001.zip › pathogens-1237913-supplementary.jpeg]
